# Household food insecurity is associated with abdominal but not general obesity among Iranian children

**DOI:** 10.1186/s12889-017-4262-3

**Published:** 2017-04-21

**Authors:** Fateme Jafari, Simin Ehsani, Azadeh Nadjarzadeh, Ahmad Esmaillzadeh, Mahmood Noori-Shadkam, Amin Salehi-Abargouei

**Affiliations:** 1 Nutrition and Food Security Research Center, Shahid Sadoughi University of Medical Sciences, Yazd, Iran; 2 Department of Nutrition, School of Public Health, Shahid Sadoughi University of Medical Sciences, Yazd, Iran; 3 Food Security Research Center, Isfahan University of Medical Sciences, Isfahan, Iran; 4 Department of Community Nutrition, School of Nutrition and Food Science, Isfahan University of Medical Sciences, Isfahan, Iran; 5 Department of Pediatrics, Mother and Newborn Health Research Center, Shahid Sadoughi University of Medical Sciences, PO Code 8915173160 Yazd, Iran

**Keywords:** Food security, Abdominal obesity, General obesity, Children, Radimer/Cornell questionnaire

## Abstract

**Background:**

Childhood obesity is increasing all over the world. Food insecurity is mentioned as a possible risk factor; however, previous studies have led to inconsistent results in different societies while data are lacking for the Middle East. We aimed to investigate the relationship between food insecurity and general or abdominal obesity in Iranian children in a cross-sectional study.

**Methods:**

Anthropometric data including height, weight, and waist circumference were measured by trained nutritionists. General and abdominal obesity were defined based on world health organization (WHO) and Iranian reference curves for age and gender, respectively. Radimer/Cornell food security questionnaire was filled by parents. Data about the physical activity of participants, family socio-economic status, parental obesity and data about perinatal period were also gathered using self-administered questionnaires. Logistic regression was incorporated to investigate the association between food insecurity and obesity in crude and multi-variable adjusted models.

**Results:**

A total of 587 children aged 9.30 ± 1.49 years had complete data for analysis. Food insecurity at household level was significantly associated with abdominal obesity (odds ratio (OR) = 1.54; confidence interval (CI):1.01–2.34, *p* <0.05) and the relationship remained significant after adjusting for all potential confounding variables (OR = 2.02; CI:1.01–4.03, *p* <0.05). Food insecurity was associated with general obesity neither in crude analysis and multi-variable adjusted models.

**Conclusions:**

The slight levels of food insecurity might increase the likelihood of abdominal obesity in Iranian children and macroeconomic policies to improve the food security are necessary. Large-scale prospective studies, particularly in the Middle East, are highly recommended to confirm our results.

## Key Messages

- Food insecurity is mentioned as a possible risk factor for over-nutrition among children.
- Previous studies on the association between food insecurity and overweight/obesity have led to inconsistent results while data are lacking from Middle East.
- The present study revealed that food insecurity at household level might increase the likelihood of abdominal obesity in Iranian children.
- Food insecurity was not associated with childhood general overweight or obesity in this sample of Iranian Children.

## Background

The worldwide prevalence of childhood obesity has been increased in recent decades. Statistics show that the prevalence of childhood overweight around the world ranges 5.1 – 24.5% in different areas [1]. Although studies have shown that the developed countries have greater pace in the childhood obesity growth compared to low and middle-income countries [2], it is estimated that the prevalence of childhood overweight or obesity also has been increased in developing countries (from about 8.1% in 1980 to 12.9% in 2013 for boys and from 8.4 to 13.4% in girls) [3].

Different environmental factors have been linked to childhood obesity. Food insecurity is also proposed to be associated with childhood overweight and/or obesity by a number of studies in recent years [4]. World Health Organization (WHO) have defined food security “when all people at all times have access to sufficient, safe, nutritious food to maintain a healthy and active life” [5]. Food insecurity is more common in developing countries. Some of the reasons may be rapid population increases, slow and variable growth in domestic food production, the limited financial capacity to import food, lack of efficient food markets, low household income, limited use of agricultural inputs and inadequate rural infrastructure [6]. Both food insecurity and obesity are emerging public health concerns in these countries. The possible association between food insecurity and obesity might be explained by limited variety of food available, compensating the food shortages by eating cheaper but more energy-dense food [7], fewer fruits and vegetables consumption [8], lack of micronutrients intake [9], behaviors like hiding foods, binge eating, and night-time eating in food-insecure children [10] and changes in the food pattern, for example, more intake of high-fat foods to prevent hunger [7] in food insecure people. Nevertheless, studies trying to find the association between food insecurity and obesity in different societies have reached to inconsistent results. For instance, several studies could not find a statistically significant difference in the prevalence of obesity between food secure and insecure populations from United States and Trinidad and Tobago [11, 12]. On the other hand, a survey from Finland indicated that the association between food insecurity and obesity might be curvilinear [13], and a number of studies suggested that the food insecure children are more likely to be obese than those who were food secure [14, 15]. In contrast, there are several pieces of evidence that revealed an inverse association between food insecurity and obesity among children [16, 17].

As there are limited data on the association between food insecurity and childhood obesity particularly from the Middle East and importance of obesity in this age group, in the present study we tried to examine the association between food insecurity and childhood obesity in a sample of Iranian primary school children residing in Isfahan, central Iran.

## Method

### Study design and population

**Fig. 1:**
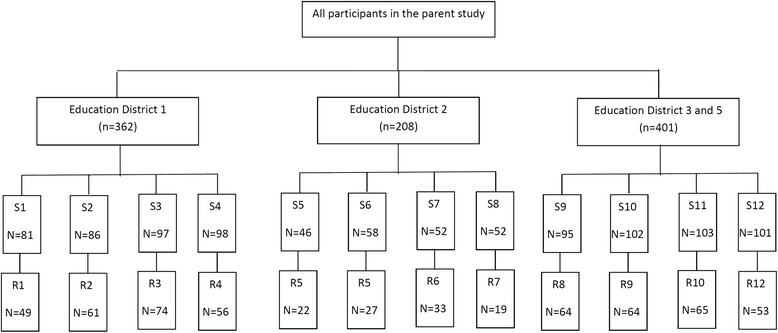
Clusteresd sampling procidure used to select the study participants from each school (S1-12) for the parrent study as well as participants who were eligible and remained to be included in the current study (R1-12)

The present cross-sectional study was carried out among a sample of 971 children (aged 7–12 years) attending 12 elementary schools, from three geographical areas (four educational destricts) of Isfahan, Iran in 2014. The selection process of participants is completely described elsewhere [18]. After exclusion of participants with special dietary restrictions, prohibition or restriction of food intake in the previous year (*n* = 50), congenital diseases (*n* = 2), and not having complete data on food insecurity, anthropometric measures, or covariates (*n* = 332), 587 participants remained for the current analysis. Clusteresd sampling procidure used to select the study participants from each school for the parrent study as well as participants who were eligible and remained to be included in the current study is illustrated in Fig. 1. There was no statistical difference between excluded and included participants based on available data like age, gender, BMI and waist circumference (WC). The study protocol was approved by the ethics Committee of Isfahan University of Medical Sciences and informed consents for entering the study and publication of study results was taken from each participant’s parents.

### Anthropometric measurements

Trained nutritionists measured the weight of students with minimum clothes and by using a digital scale to the nearest 0.1 kg. Measurement of height was conducted according to standard procedures in a standing position without shoes by using a plastic tape measure fixed on a wall to the nearest centimeter. We calculated body mass index (BMI) dividing weight in kilogram by height squared in the meter. Waist circumference (WC) was assessed by using a non-stretchable plastic tape placed between the iliac crest and the last rib with an accuracy of 0.5 cm when the participants were standing. All anthropometric measurements were conducted from 8 to 12 a.m for three times and mean values were recorded.

BMI values above the 95th percentile of BMI for age and gender, defined by WHO [19] and WC values higher than the 75th percentile of waist circumference for age curves defined for Iranian children [20] were considered as general and central obesity, respectively.

### Assessment of food insecurity

Food insecurity was assessed using Radimer/Cornell questionnaire which was filled by parents. It is supposed that this tool is able to capture most of the food insecurity components. In this study, we used a self-reported modified version of Radimer/Cornell food insecurity questionnaire which its validity and reliability were confirmed to be used in Iran [21]. The questionnaire consisted of 16 questions asked for experiencing food insecurity at three levels: family, adult, and child (8, 5, and 3 questions for each level, respectively). Each question had three possible answers: “not true”, “sometimes true” and “often true”. Participants in the present study were categorized based on their food insecurity level into four groups: Household food secure, Household food insecure, Individual food insecure, and Child hunger. People who can not afford to buy good food for a family are household food insecure, but in individual food insecurity and child hunger levels, they even can not provide enough food for themselves and their children, respectively [21]. In the present study, if all questions were answered as “not true”, participants were placed at the household food secure level; If parents answered that “sometimes” or “often” experienced one or more situations explained in questions designed for household part of the questionnaire and answered “not true” to the rest of questions, their child was placed at household insecure. If they answered “sometimes true” or “often true” to one or more questions designed for adult individual items, their child was categorized as adult insecure and if parents answered “sometimes true” or “often true” to one or more questions of child food security items the participant was assigned to child hunger level. The food insecurity level increases from household food insecurity to child hunger.

### Assessment of covariates

Parents also completed a self-administered questionnaire, which asked about each participants’ maternal age, family size, parental education (University educated/graduate from high school or lower) and obesity (obese or normal).

Information about participants’ perinatal period including birth weight, birth order, length of gestation (preterm/normal/post-term), multiple birth (yes/no), breastfeeding status (exclusive/nonexclusive) and initiation of complementary feeding (before or after 6 months of age) were also obtained through self-reported questionnaires completed by the mothers.

Physical activity was evaluated by using a validated Physical Activity Questionnaire for Iranian Children (PAQ-C) [22] that was completed by parents. The participants were classified into three groups based on scores from the questionnaire: Inactive, semi-active, active.

To realize the economic status of families, we used a questionnaire that contained 15 questions evaluating parents’ occupation, household income, family head (father/other family members), ownership status of house, number and model of cars owned by family, number of bedrooms and facilities inside home (including furniture, microwave, dishwasher, carpet, washing machine, laptop, or personal computer). We used multiple correspondance analysis to combine these categorical variables to derive the overal economic status score as a numerical variable [23]. Then the participants were placed into low, moderate, and wealthy groups based on tertiles of total economic status scores.

### Statistical analysis

As the present study was used clustered sampling method to select the study participants, we checked the the intraclass correlation (ICC) coefficient for age standardized BMI and WC defining schools as random factor using general linear model. Comparison of continuous and categorical variables across participants’ food insecurity level was done using analysis of variance (ANOVA) with Bonferroni post hoc test and chi-square test, respectively. We used binary logistic regression to assess food insecurity in relation to general and abdominal obesity in univariate and several multivariate models. In the first model, the adjustment was performed for age and gender. In second model, the perinatal variables possibly associated with childhood obesity including length of pregnancy (preterm/term), birth weight (in grams), birth order, multiple birth (yes/no), breastfeeding status (exclusive/non-exclusive), initiation of complementary feeding, length of breastfeeding, and maternal age at birth were adjusted besides the items adjusted in the first model. The third model was obtained by further adjustment for mother education, father education, mother obesity, father obesity, household economic status, and physical activity of the child. Data were analyzed by using the Statistical Package for Social Sciences (SPSS version 16). *P* values <0.05 were considered a statistically significant.

## Results

Complete data were available for 587 children (439 girls and 148 boys) aged 9.30 ± 1.49 years’ of which 28.8% were household food secure, 33.4% were household food insecure, 19.8% were food insecure at individual level, and 18.1% experienced child hunger. Our analysis on intra class corrlation (ICC) revealed that the ICC coefficients for schools were not high (0.005 and 0.043 for age standardized BMI and WC, respectively).

Participants’ general characteristics based on food security levels are summarized in Table 1. Participants’ age, gender, BMI, general obesity, physical activity, prenatal characteristics such as multiple birth, length of pregnancy, or age of mother at pregnancy, and birth characteristics including birth weight, birth order, exclusive breastfeeding, complementary feeding onset and length of breastfeeding was not statistically different in food insecurity levels. In contrast, waist circumference and abdominal obesity were positively associated with food insecurity (*P* < 0.05). Parental obesity was directly related to the severity of food insecurity so that by increasing severity of food insecurity from household food secure to child hunger, the prevalence of mother’s obesity significantly increased and this association was marginally significant for fathers (Table 1). However, there was an inverse relationship between the level of parents’ education and food insecurity. Our analysis revealed that the family size increases as the food security worsens. There was also an inverse relationship between socioeconomic status and food insecurity (Table 1).

**Table 1:** General characteristics of the study population based on food insecurity levels

	Household food secure (*n* = 169)	Household food insecure (*n* = 196)	Individual food insecure (*n* = 116)	Child hunger (*n* = 106)	*P*-value
Age (year)	9.19 ± 1.43^a^	9.30 ± 1.52	9.34 ± 1.47	9.44 ± 1.56	0.593
BMI (Kg/m^2^)	17.51 ± 3.14	17.59 ± 3.83	17.20 ± 3.74	18.32 ± 12.19	0.577
Waist circumference (cm)	62.57 ± 8.38	64.57 ± 9.25	61.52 ± 9.25	64.15 ± 9.66	0.02
Family size (number)	3.97 ± 0.82	3.98 ± 0.87	4.15 ± 0.89	4.39 ± 1.66	0.01
Birth order	1.62 ± 0.88	1.65 ± 0.87	1.66 ± 0.79	1.91 ± 1.20	0.06
Birth weight (gram)	3046.16 ± 681.23	2974.82 ± 794.44	3089.09 ± 2404.24	2729.67 ± 1097.59	0.16
Maternal age at birth (year)	26.23 ± 5.08	26.79 ± 4.83	26.24 ± 4.98	26.55 ± 5.78	0.71
Gender (female) (%)	72.2	76	74.1	77.4	0 .76
Physical activity (%)					
Low	31	39.4	30.1	35.6	0.26
Moderate	27.6	32.5	35.5	27.6	
Severe	41.4	28.1	34.4	36.8	
Obesity (%)					
Abdominal obesity	38.5	48.4	25.9	39.2	0.002
General obesity	20.1	22.4	12.9	14.2	0.109
Multiple births (%)	1.8	1.5	0.9	3.8	0.41
Preterm birth (%)	10.4	9.5	11.9	16.8	0.28
Exclusive breastfeeding (%)	59.1	56.5	52.8	52.9	0.67
Commencement of complementary feeding before 6 months (%)	17.9	13.4	21.2	15.2	0.32
Breastfeeding under 6 month (%)	8.5	9.5	6.4	11.5	0.61
Mother education (higher than high school diploma) (%)	52.8	42.6	17.4	15.2	<0.001
Father education (higher than high school diploma) (%)	60.5	39.7	20.4	17.2	<0.001
Obese Mother (%)	11.8	11.1	10.9	24.0	0.03
Obese Father (%)	11.4	13.3	8.2	21.6	0.07
Economic status (%)					
Low	10.1	21.0	49.5	77.4	<0.001
Moderate	28.8	40.7	34.1	17.9	
Wealthy	61.2	38.3	16.5	4.8	

^a^Values are mean ± standard deviation, otherwise indicated

Our analysis could not show a significant association between food insecurity and odds of general obesity. After adjustment for confounding variables, the association remained non-significant (Table 2). In contrast, children who were household food insecure had higher chance for being abdominally obese compared to household food secure participants (OR = 1.54, 95% CI: 1.01–2.34). When the association was additionally adjusted for age, gender and variables related to the birth characteristics in the second model, this relationship remained significant (OR = 1.97, 95% CI: 1.23–3.15). The association was even after adjustment for other variables such as mother education, father education, mother obesity, father obesity, household economic status, and physical activity (OR = 2.02, 95% CI: 1.01–4.03) (Table 2). Furthermore, children who lived in families with individual food insecurity level had less odds of having abdominal obesity than their household food secure counterparts (OR = 0.57, 95% CI: 0.34–0.97) and this association remained significant after adjustment for age and gender (OR = 0.54, 95% CI: 0.32–0.93). However, further adjustment for perinatal characteristics and variables related to the child's family, changed the association into non-significant (Table 2).

**Table 2:** Odds ratios and 95% confidence intervals (CIs) for the association between food insecurity and obesity

	Household food secure OR (95% CI)	Household food insecure OR (95% CI)	Individual food insecure OR (95% CI)	Child hunger OR (95% CI)
General Obesity				
Crude	1	1.04 (0.66–1.64)	0.86 (0.50–1.48)	0.84 (0.48–1.47)
Model 1^a^	1	1.05 (0.66–1.67)	0.86 (0.50–1.48)	0.85 (0.49–1.50)
Model 2^b^	1	1.34 (0.81–2.23)	0.93 (0.50–1.70)	0.88 (0.47–1.63)
Model 3^c^	1	1.04 (0.49–2.24)	0.85 (0.31–2.31)	0.53 (0.16–1.68)
Abdominal Obesity				
Crude	1	1.54 (1.01–2.34)*	0.57 (0.34–0.97)*	1.05 (0.64–1.75)
Model 1^a^	1	1.49 (0.97–2.29)	0.54 (0.32–0.93)*	0.99 (0.59–1.65)
Model 2^b^	1	1.97 (1.23–3.15)**	0.60 (0.33–1.08)	0.93 (0.52–1.63)
Model 3^c^	1	2.02 (1.01–4.03)*	1.12 (0.45–2.81)	1.44 (0.54–3.86)

*P value < 0.05 ** *P* value < 0.01

^a^Adjusted for age and gender

^b^Adjusted for variables in Model 1 plus birth Characteristics such as birth weight, birth order, multiple birth, exclusivity of breast feeding, complementary feeding, length of gestation, length of breastfeeding, maternal age at birth

^c^Adjusted for variables in Model 2 plus mother education, father education, mother obesity, father obesity, household economic status, and physical activity of children

## Discussion

The present cross-sectional study examined the associations between food insecurity with childhood general and abdominal obesity in a sample of Iranian children and demonstrated that there is a significant association between food insecurity at household level and abdominal obesity after adjusting maximum number of possible confounders; however, more severe levels of food insecurity was not associated with abdominal obesity. Food insecurity also was not related to likelihood of general obesity.

To the best of our knowledge a limited number of studies have been conducted trying to find the association between food insecurity and childhood obesity particularly in Middle East. We could find only two studies with limited number of participants in Iranian children [24, 25]. Karam soltani et al. [25] conducted a case–control study in yazd provience on 394 obese (cases) and non-obese (controls) students between 9 and 11 years old. The prevalence of food insecurity were assessed by using USDA food security questionnaire in the case and control group that were 30.5 – 35.2% respectively and they did not observe any significant differences in the prevalence of food insecurity between the two groups. Basirat et al. [24] also in a cross sectional study on 314 students with 6 – 11 years old from Farokhshahr discovered that 69.4% of households suffered from moderate to severe food insecurity based on information obtained from Radimer/Cornell questionnaire but did not find a significant association between food insecurity and BMI or abdominal obesity. In fact, we could include more children in the present study and also we adressed the abdominal obesity while the previous investigations among the Iranian children did not assessed the association for abdominal obesity.

Several studies have tried to find the association between food security and obesity, worldwide. For instance, a study done in 5–12 years old children in the United States, found a significant association in girls, but this relationship was not seen among boys [26]. A study carried out in 2,516 American children aged 7 – 16 years old, did not find any association between food insecurity and obesity [27]. In contrast, inverse relationship was observed between overweight and poverty after controlling for several factors (age, gender, and educational status of parents) in a study carried out by Hofferth et al. among children residing in United States [28]. On the other hand, a study conducted in the context of national health and nutrition examination survey revealed an increasing trend in general and abdominal obesity in adolescents aged 12 – 18 years [29]. In Bogota, the prevalence of underweight in food insecure children was three times higher than food secure children [16]. The previous results published in this regard, showed an equivocal relationship between food insecurity and obesity and this might be because of the difference in questionnaires used to assess food security, study designs and the availability of low cost high energy foods in the region. In the present study although children with household food insecurity had higher odds for general obesity, we could not find a significant association; however, the association was significant for abdoinal obesity. While the results were inconsistent [30] a number of previous studies have proposed that waist circumference might be a more sensitive marker for obesity compared to BMI [29] and this might explain the association found for abdominal obesity. The increased chance of abdominal obesity that we found in our study, might be because of the nutritional transition in Iran which is accompanied by urbanization, population growth, major changes in diet, and declined physical activity [31].

As a general assumption, food insecurity might increase the likelihood of obesity due to the following reasons: increased consumption of inexpensive energy-dense foods [32], eating too much at times when food is abundant [33], metabolic changes to ensure more efficient use of energy [34], different standards for a healthy diet [35], parents more feed their children to protect them at the time of food abundance [36], and ultimately existence of food insecurity during pregnancy [37].

It can be realized that food insecurity is likely due to less protein intake [38], the inability to preparation and use of fruits, vegetables [39–41] and dairy [42] groups and finally high consumption of low price fatty and sugary foods [41] is more associated with abdominal obesity rather than general obesity.

Our study had some limitations that should be considered. In the present study we lost 384 participants. A large number of study participants had refused to answer the food insecurity questionnaire. Although the loss of participants might have affected our results, we could not find any statistical difference between participants who were excluded from analyses and they included regarding age, gender, BMI and waist circumference. Due to the small sample size in each of the two sexes, we could not explore the possible gender specific associations. In addition, casual relationship between food insecurity and obesity cannot be inferred using retrospective observational studies; therefore, conducting prospective studies is highly recommended.

## Conclusion

In conclusion, the present cross-sectional study revealed that mild levels of food insecurity might increase the likelihood of abdominal obesity in Iranian children. More studies with prospective design are needed to confirm our results. It is suggested that social policies affect food security [43] and policy makers have to take this point into account and also try to improve the situation by considering macroeconomic policies to improve the food security are necessary.
